# Aortic dilatation and gastrointestinal disease: expansion of the hyperinsulinism-polycystic kidney disease phenotype

**DOI:** 10.1210/jendso/bvag243

**Published:** 2026-10-21

**Authors:** Daphne Yau, Unai Diaz-Moreno, Elena Cervi, Meera Shaunak, Ereny Bassilious, Jonathan Kraus Friedmann, Craig Jefferies, Clare Gilbert, Kate Morgan, Nicola Townsend, Stephen Marks, Jelena Stojanovic, Iona Madden, Detlef Bockenhauer, Fevronia Kiparissi, Kelsey Jones, Stephanie Grunewald, Sarah E Flanagan, Antonia Dastamani

**Affiliations:** Division of Endocrinology, Department of Pediatrics, University of Saskatchewan, Saskatoon S7N 0W8, Canada; Department of Metabolic Medicine, Great Ormond Street Hospital for Children NHS Foundation Trust, London WC1N 3JH, UK; Centre for Inherited Cardiovascular Diseases, Cardiology Department, Great Ormond Street Hospital for Children NHS Foundation Trust, London WC1N 3JH, UK; Department of Paediatric Endocrinology, Great Ormond Street Hospital for Children NHS Foundation Trust, London WC1N 3JH, UK; Division of Endocrinology, Department of Pediatrics, McMaster University, Hamilton L8S 4L8, Canada; Universidad de Chile, Santiago, Chile Pediatric Endocrinology Unit, Luis Calvo Mackenna Children´s Hospital and Clinica Alemana, Santiago 7500539, Chile; Starship Children's Health, Heath New Zealand- Te Whatu Ora Te Toka Tumai, Auckland 1023, New Zealand; Department of Paediatric Endocrinology, Great Ormond Street Hospital for Children NHS Foundation Trust, London WC1N 3JH, UK; Department of Paediatric Endocrinology, Great Ormond Street Hospital for Children NHS Foundation Trust, London WC1N 3JH, UK; Department of Paediatric Nephrology, Great Ormond Street Hospital for Children NHS Foundation Trust, London WC1N 3JH, UK; Department of Paediatric Nephrology, Great Ormond Street Hospital for Children NHS Foundation Trust, London WC1N 3JH, UK; NIHR Great Ormond Street Hospital Biomedical Research Centre, University College London Great Ormond Street Institute of Child Health, London WC1N 1EH, UK; Department of Paediatric Nephrology, Great Ormond Street Hospital for Children NHS Foundation Trust, London WC1N 3JH, UK; University College London Great Ormond Street Institute of Child Health, London WC1N 1EH, UK; Department of Paediatric Nephrology, Great Ormond Street Hospital for Children NHS Foundation Trust, London WC1N 3JH, UK; Paediatric Nephrology Unit, Bordeaux University Hospital, Centre de Référence Maladies Rénales Rares Sorare, Bordeaux 33076, France; Department of Paediatric Nephrology, Great Ormond Street Hospital for Children NHS Foundation Trust, London WC1N 3JH, UK; Paediatric Nephrology, University Hospital Leuven and PKD Research Group, Department of Cellular and Molecular Medicine, KUL, Leuven BE-3000, Belgium; Department of Paediatric Gastroenterology, Great Ormond Street Hospital for Children NHS Foundation Trust, London WC1N 3JH, UK; Department of Paediatric Gastroenterology, Great Ormond Street Hospital for Children NHS Foundation Trust, London WC1N 3JH, UK; Department of Metabolic Medicine, Great Ormond Street Hospital for Children NHS Foundation Trust, London WC1N 3JH, UK; Clinical and Biomedical Science, University of Exeter, Exeter EX2 5DW, UK; Department of Paediatric Endocrinology, Great Ormond Street Hospital for Children NHS Foundation Trust, London WC1N 3JH, UK

**Keywords:** *PMM2*, phosphomannomutase 2, hyperinsulinism, polycystic kidney disease, inflammatory bowel disease

## Abstract

**Context:**

Congenital hyperinsulinism (HI) with polycystic kidney disease (PKD), or HIPKD, is a recently described syndrome caused by a specific promotor variant in *PMM2*, a gene otherwise associated with PMM2 congenital disorder of glycosylation.

**Objective:**

To describe the HIPKD clinical phenotype, expanding our understanding and its distinction from the congenital disorder of glycosylation phenotype.

**Design:**

Retrospective multicenter cohort study of individuals with HIPKD identified through an international HI genetic referral center.

**Patients:**

Ten individuals from 9 families compound heterozygous for the promoter variant c.-167G > T and a pathogenic intragenic variant in *PMM2*.

**Intervention:**

None.

**Main Outcome Measures:**

Observed clinical features and outcomes.

**Results:**

Median age of presentation was 5 months (range 0-42). Median age at follow up was 12.7 years (range 5.4-20.1). HI was managed with diazoxide monotherapy (7/10 patients), diazoxide and nifedipine (2/10 patients), and carbohydrate supplementation alone (1/10 patient). PKD age at diagnosis and severity was variable, ranging from antenatal presentation requiring renal transplantation, to incidental detection in mid-childhood. Liver cysts were noted in 6/10 patients (5/6 with normal liver function). Five of 7 patients had systemic hypertension and 3/5 were normotensive on losartan at last follow up. Inflammatory bowel disease (IBD) with gastric antral foveolar hyperplasia occurred in 4/10 patients, and aortic root and/or ascending aortic dilation in 4/7 patients who underwent echocardiography, 3 of whom also had IBD.

**Conclusion:**

HIPKD due to a *PMM2* promoter variant is associated with broad, multisystem phenotype, including IBD and aortic dilation. These findings support targeted screening for IBD and cardiovascular surveillance in addition to standard endocrine and renal follow up.

Glycosylation is a key biological process by which proteins and lipids are modified by the addition of glycans; this process is critical in maintaining the structure and function of numerous biological molecules (1). Consequently, human disease results when genes in these pathways are disrupted and are collectively known as congenital disorders of glycosylation (CDG). The most common CDG results from pathogenic variants in *PMM2,* the gene encoding phosphomannomutase 2, a key enzyme in N-glycosylation, which is inherited in an autosomal recessive manner (1). *PMM2*-CDG can present from infancy into adulthood with a high degree of variability in clinical manifestations and disease course, with severity ranging from lethality in the first year of life to adults who are mildly affected (2). The phenotype is predominantly characterized by neurodevelopmental impairment, such as developmental delay, hypotonia, and seizures, with multisystem involvement in a subset of individuals including gastrointestinal/liver, musculoskeletal, ophthalmologic, hematologic, cardiovascular, respiratory, renal, and endocrine involvement (3).

Recently, however, a promoter variant in *PMM2,* either homozygous or occurring in trans with a pathogenic coding variant, has been described in individuals with cooccurrence of hyperinsulinism (HI) and polycystic kidney disease (PKD), a syndrome named HIPKD (4). The observed phenotype of HIPKD was distinct from PMM2-CDG, without its typical diagnostic or clinical features, most notably neurologic involvement. Rather, these individuals presented with hyperinsulinemic hypoglycemia and PKD of variable severity in infancy and early childhood, and a subset of these individuals also had hepatic cysts, which can be associated with PKD (4).

Since its initial description, further cases of HIPKD have been reported (5-7). Moreover, additional features have been described, expanding our understanding of the HIPKD phenotype. A distinctive form of inflammatory bowel disease (IBD) was described in 3 patients, including 2 individuals from the original report, with gastric antral involvement that is rare in children (8). However, data on long-term outcomes and the broader multisystem phenotype, particularly cardiovascular involvement, remain limited.

We have therefore analyzed the follow-up data of our cohort as well as more recently diagnosed cases to better delineate the phenotype and natural history of HIPKD and define the prevalence and characteristics of gastrointestinal and cardiovascular manifestations associated with the *PMM2* promoter variant.

## Methods

### Participants and clinical definitions

Seven of 10 subjects (ID 1-3, 5-7, 9) in the current study have been previously described; we report here their further clinical evolution (4, 8). In brief, 10 patients from 9 families were referred for HI genetic testing to the Exeter Genomics Laboratory, UK; including 1 patient referred via the Open Hyperinsulinism Genes Project (9). HI was diagnosed based on a detectable insulin level with a concurrent blood glucose of <3 mmol/L. PKD was clinically diagnosed based on the presence of enlarged kidneys with multiple cysts on imaging. Terminology for kidney disease was used according to the Kidney Disease: Improving Global Outcomes guidelines (10). Z-score calculations for aortic diameters were calculated as previously described for echocardiogram and magnetic resonance imaging (MRI) (11, 12).

### Data collection and statistical analysis

For all participants, data were obtained from the clinical details submitted at the time of referral for genetic testing, as well as from the referring clinician when followed up for clinical details. If estimated glomerular filtration rate was not provided by the referring clinician, it was calculated based on the Chronic Kidney Disease in Children U25 equation using serum creatinine, sex, age, and height (13). Descriptive statistics are presented as medians with ranges; no formal hypothesis testing was performed given the small sample size.

### Genetic analysis

For the 3 previously unreported patients, targeted next-generation sequencing of 22 genes associated with HI was performed on leukocyte DNA using a custom capture panel (Twist Bioscience). This included analysis of the coding regions and promoter of *PMM2* and copy number variant analysis (14). Sequencing was carried out on an Illumina Nextseq 500 platform. Parental samples were subsequently tested by Sanger sequencing to confirm compound heterozygosity of the identified variants.

### Ethical considerations

All subjects and/or their legal guardians gave informed consent for genetic testing and inclusion in this publication. In Exeter, the genetic studies were approved by the Wales Research Ethics Committee 5 (22/WA/0268), with all participants recruited to the Genetic Beta Cell Research Bank (IRAS: 316050).

## Results

Ten patients from 9 families with cooccurrence of HI and PKD were identified. Two individuals were siblings. All patients were compound heterozygous for the promoter variant c.-167G > T and a pathogenic missense variant in *PMM2* (Table 1). The age at presentation ranged from 0 to 42 months, with a median of 5 months. In terms of the initial presenting feature, 3/10 patients presented with HI, 4/10 patients presented with PKD, and 3/10 patients presented concurrently with HI and PKD. Median age at follow-up was 11.9 years (range 5.4-20.1 years).

**Table 1:** Genetics and details of hyperinsulinism and kidney and liver disease

Patient ID	1*^a^*	2*^a,b^*	3*^a,b^*	4	5*^b^*	Siblings		8	9*^b^*	10
						6*^b^*	7*^b^*			
PMM2 Variant										
Nucleotide	c.-167G > T/c.442G > A	c.-167G > T/c.422G > A	c.-167G > T/c.422G > A	c.-167G > T/c.422G > A	c.-167G > T/c.422G > A	c.-167G > T/c.422G > A	c.-167G > T/c.422G > A	c.-167G > T/c.422G > A	c.-167G > T/c.422G > A	c.-167G > T/c.422G > A
Predicted protein	p.?/p.Asp148Asn	p.?/p.Arg141His	p.?/p.Arg141His	p.?/p.Arg141His	p.?/p.Arg141His	p.?/p.Arg141His	p.?/p.Arg141His	p.?/p.Arg141His	p.?/p.Arg141His	p.?/p.Arg141His
Ethnicity	Caucasian	Caucasian	Caucasian	Caucasian	Caucasian	Caucasian	Caucasian	Hispanic	Caucasian	Caucasian
Age at last follow up (years)	10.6	12.7	11.2	20.1	15.3	17.2	16.2	8	11	10.7
Hyperinsulinism										
Sex	Male	Male	Male	Male	Female	Male	Female	Male	Female	Male
Birth weight (g)	4100	4250	2960	3487	3770	3400	3850	2000	4160	4390
% for gestational age	94.8	99.4	38.6	60	88.7	65.2	91.7	76.3	97.9	98.3
Age at diagnosis (months)	18	0	0	17	13	8	42	48	18	0
Medication	Diazoxide Nifedipine	Diazoxide	Diazoxide Nifedipine	Diazoxide	None	Diazoxide	Diazoxide	Diazoxide	Diazoxide	Diazoxide
Carbohydrate supplementation	No	Yes	Yes	Yes	Yes	No	No	Yes	Yes	No
Management at follow up	Diazoxide	Diazoxide	Diazoxide	None	None	Diazoxide	Diazoxide	Diazoxide	Diazoxide	Diazoxide
Kidney Disease										
Age at diagnosis (months)	18	Antenatal	0	120	36	0	48	8	2	Antenatal
eGFR at diagnosis (mL/min/1.73 m^2^)	N/A	20	50	N/A	95	90	95	75	120	N/A
Kidney size	Enlarged	Enlarged	Enlarged	Enlarged	Enlarged	Enlarged	Enlarged	N/A	Enlarged	Enlarged
Morphology	Cysts	Cysts	Cysts	Cysts	Cysts	Cysts	Cysts	Cysts	Cysts	Cysts
eGFR at follow up	93.5	16.3 Post-transplant	76.5	N/A	91.2	53.4	93	63	141	48 Post-transplant
Liver Disease										
Ultrasound	Normal	Cysts	Normal	Fatty liver	Normal	Cysts	Cysts	Cysts	Cysts	Cysts
Liver function tests	Normal	Normal	Normal	Abnormal	N/A	Normal	Normal	Normal	Normal	Abnormal

Abbreviations: eGFR, estimated glomerular filtration rate; N/A, not available.

^*a*^Previously reported (8).

^*b*^Previously reported (4).

### Hyperinsulinism

Half of the patients were large-for-gestational age (birth weight >90th percentile), whereas the remainder were an appropriate size for their gestational age (Table 1). HI was diagnosed at a median age of 15 months (range 0-48 months). HI predominantly responded to first-line pharmacotherapy with diazoxide: 7/10 patients were managed with diazoxide monotherapy, 2/10 patients with diazoxide and nifedipine, and 1/10 patient with dietary carbohydrate supplementation alone. Of the patients on diazoxide, 4/7 patients on diazoxide monotherapy, and 1/2 patients on diazoxide along with nifedipine also received carbohydrate supplementation in their diet. None received escalation of therapy such as somatostatin receptor agonist treatment. Of the 7 patients on diazoxide monotherapy, 1 stopped diazoxide at 13 years of age with stable blood glucose levels, follow-up data were unavailable for 1 patient, and the remaining 5 patients remained on diazoxide at a median age of 12.7 years (range 5.4-17.2). The 2 patients on diazoxide and nifedipine (ID 1 and ID 3) did not respond to nifedipine monotherapy and remained on diazoxide at 10.6 and 11 years of age, respectively (Table 1).

### Polycystic kidney disease

All patients had enlarged, cystic kidneys on imaging. The age at presentation was a median of 5 months (range 0-120 months), with 2 individuals (ID 2, 10) diagnosed antenatally based on ultrasound findings (Table 1). Renal prognosis was worse in those who were diagnosed antenatally or within the first month of life (ID 2, 3, 6, 10) compared to those diagnosed after the neonatal period, with patients ID 2 and ID 10 undergoing renal transplant at 2 years of age because of progression to kidney failure (KF). Patient ID 2 received a second renal transplant at 13.3 years because of a decline in renal allograft function, which had lasted for 8 years and 8 months. Estimated glomerular filtration rate at most recent follow up for these 4 patients was a median of 57.2 mL/min/1.73 m^2^ (range 16.3-76.5) at a median 12 years of age (range 6.3-16.6) vs a median of 93 mL/min/1.73 m^2^ (range 63-141) at a median 11 years of age (range 5.4-16.2) for those diagnosed beyond the first month of life (Table 1). None of the patients diagnosed after 1 month of life progressed to KF at the time of last follow up.

### Hepatic involvement

Liver cysts were noted in 6/10 patients. Liver function testing was available in 9/10 patients and was normal in 5/6 patients with cysts in addition to 2 other patients with normal imaging. One individual with liver cysts (ID 10) had a persistent elevation in alkaline phosphatase with normal gamma-glutamyl transferase, alanine aminotransferase, and aspartate aminotransferase; at his most recent follow up, he met criteria for Caroli disease, characterized by segmental dilation of large, intrahepatic bile ducts, based on ultrasound and elastography (15). Another individual (ID 4) had evidence of hepatic steatosis and fibrosis on ultrasound accompanied by abnormal liver function.

### Inflammatory bowel disease

Four of 10 patients were diagnosed with a rare form of IBD, 3 of whom have been previously reported (8) (Table 2). Similar to the previous cases, the fourth patient (ID 6) demonstrated hyperplasia of the gastric antrum with evidence of chronic inflammation based on the presence of polyps and histology confirmed hyperplasia of the gastric antrum with chronic inflammation. Age at presentation ranged from 6 months to 16 years. However, for patient ID 2, esophagogastroduodenoscopy performed to screen for varices because of the risk of portal hypertension secondary to PKD noted the presence of gastropathy at 2 years of age; he was asymptomatic at the time and did not present with gastrointestinal symptoms until 10 years of age. Similarly, although patient ID 10 has not been diagnosed with IBD, he presented with abdominal pain at 5 years of age, and nonspecific inflammatory changes involving the colon were noted on ultrasound. At 8 years of age, he developed diarrhea and poor weight gain. Fecal calprotectin was elevated but normalized spontaneously after 7 months and he did not undergo esophagogastroduodenoscopy. The findings may represent subclinical disease but did not meet criteria for diagnosis of IBD.

**Table 2:** Inflammatory bowel disease details of patients with HIPKD

Patient ID	1*^a^*	2*^a,b^*	3*^a,b^*	4	5*^b^*	Siblings		8	9*^b^*	10
						6*^b^*	7*^b^*			
Present	Yes	Yes	Yes	N/A	N/A	Yes	N/A	No	No	No
Age at presentation (years)	0.5	10	6	N/A	N/A	16	N/A	Not applicable	Not applicable	Not applicable
Symptoms at presentation	GI bleeding	Diarrhea	Diarrhea	N/A	N/A	GI bleeding	N/A	Not applicable	Not applicable	Not applicable
Pathology	Gastric antral foveolar hyperplasia, colonic lymphonodular hyperplasia	Gastric antral foveolar hyperplasia, chronic active pancolitis	Gastric antral foveolar hyperplasia, chronic active pancolitis	N/A	N/A	Gastric antral foveolar hyperplasia	N/A	Not applicable	Not applicable	Not applicable

Abbreviations: GI, gastrointestinal; N/A, not available.

^*a*^Previously reported (8).

^*b*^Previously reported (4).

### Cardiac manifestations

Dilation of the aortic root and ascending aorta was identified in 4/7 patients who underwent an echocardiogram (Table 3). Dilation of the aorta was not detected at initial assessment but was documented during surveillance in 3/4 patients between 3.5 and 9 years of age. Dilation involved the aortic root at the level of the sinuses of Valsalva with diameters 23.6 to 33 mm and to a greater extent the ascending aorta at 22.2 to 32 mm (Table 3). There was no evidence of rapid progression during continued surveillance. None of the individuals with aortic dilatation had cardiac symptoms and the findings were incidental with echocardiograms requested for other reasons. No other cardiac or vascular abnormalities were identified on imaging including in the 2 patients (ID 1, 2) who underwent a cardiac MRI. At last follow up, there were no clinical concerns regarding cardiac status. Five of 7 patients had high blood pressure, including 3/4 patients with aortic dilation and were on treatment with losartan (Table 3).

**Table 3:** Cardiovascular features of patients with HIPKD

Patient ID	1*^a^*	2*^a,b^*	3*^a,b^*	4	5*^b^*	Siblings		8	9*^b^*	10
						6*^b^*	7*^b^*			
Age at first echocardiogram (months)	18	16	5	N/A	N/A	N/A	N/A	N/A	N/A	37
Echocardiogram at detection of aortic dilation										
Age (years)	3.5	9	9	N/A	No aortic dilation	No aortic dilation	N/A	No aortic dilation	N/A	6.3
SoV size (mm)					Not applicable	Not applicable		Not applicable		23.6
SoV Z-score	2.4	5.3	3.2							2.3
AA size (mm)										22.2
AA Z-score	3.9	7.9	5.4							3.3
Most recent echocardiogram										
Age (years)	10.9	15.4	10	N/A	Not applicable	Not applicable	N/A	Not applicable	N/A	10.2
SoV size (mm)	28.8	32.9	29							26.2
SoV Z-score	3.7	3.1	3.2							3.5
AA size (mm)	27.5	32	28							26.1
AA Z-score	4.5	4.2	4.4							5
Cardiac MRI										
Age (years)	11	9	N/A	Not applicable	Not applicable	Not applicable	Not applicable	Not applicable	Not applicable	N/A
SoV size (mm)	27	35								
SoV Z-score	2.5	6.3								
AA size (mm)	26	31								
AA Z-score	3.8	6.8								
Elevated blood pressure										
Present	Yes	Yes	Yes	Yes	No	Yes	No	Yes	No	No
Medications	losartan	losartan	losartan	N/A	Not applicable	No	N/A	amlodipine, enalapril	Not applicable	No

Abbreviations: AA, ascending aorta; N/A, not available; SoV, sinuses of Valsalva.

^*a*^Previously reported (8).

^*b*^Previously reported (4).

### Neurodevelopmental manifestations

Neurodevelopmental concerns were noted in 4/9 patients; data were unavailable for 1 individual. One patient developed absence epilepsy at 9 years of age (ID 5), 2 patients (ID 1, 2) reported attentional difficulties with attention deficit hyperactivity disorder (ADHD) formally diagnosed in 1 patient, and a fourth patient reported possible learning difficulties (ID 6). The patient diagnosed with ADHD underwent brain MRI at 2 years of age, which detected mild, nonspecific signal changes in the bilateral subcortical posterior occipital white matter but no structural abnormalities.

## Discussion

We describe a case series of 10 individuals with an ultrarare disorder, HIPKD, due to compound heterozygosity for promoter and missense variants in *PMM2*. A subset of these were identified to have unique gastrointestinal and cardiac involvement during follow-up expanding the associated phenotypic spectrum. Four of 10 individuals developed a rare form of IBD involving the gastric antrum, unusual in the location of involvement occurring in childhood. Three of 4 of these individuals were also found to have dilatation of the aorta, as did 1 additional patient who did not have IBD. These features have not been reported by other groups in individuals with the *PMM2* promoter variant despite a follow-up duration that varied from months to several decades (5-7, 16) (Fig. 1). Similar to previous reports of HIPKD, the HI and PKD features presented in infancy and early childhood, except for 1 patient who presented with kidney disease at 10 years of age (Table 1). The HI was manageable with the first-line medication, diazoxide, with some patients receiving additional dietary carbohydrate supplementation and/or adjunctive treatment with the calcium channel blocker, nifedipine. The PKD severity varied from antenatal presentation with KF in early childhood to incidental finding in childhood. Liver cysts occurred in just over half with normal liver function in all except for 2 patients, 1 with liver cysts, Caroli disease, and isolated alkaline phosphatase elevation, and the other with fatty liver on ultrasound.

**Figure 1:**
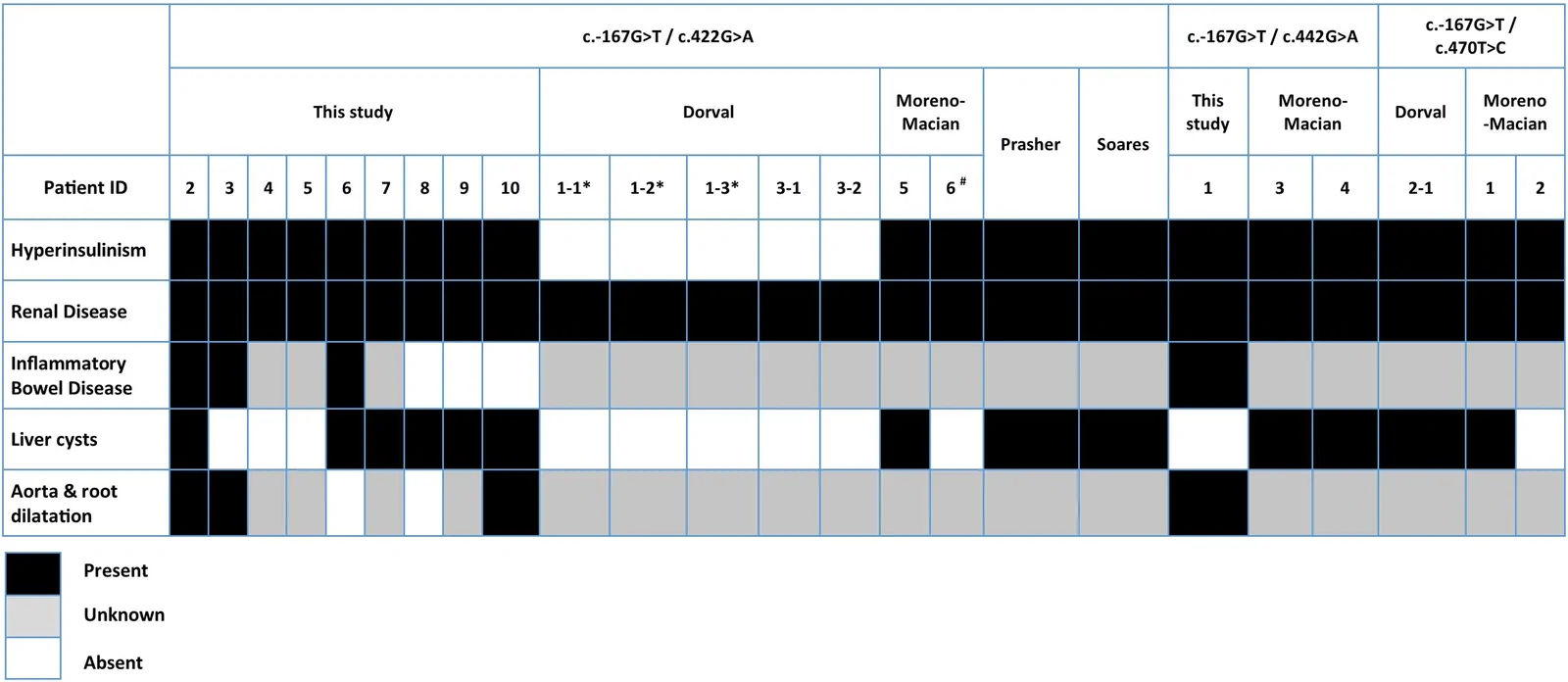
Comparison of features in cases of HIPKD. Comparison between the clinical features of the patients in this study and previously reported cases (5-7, 16). Cases were grouped by genotype. Features reported as present are shaded in gray, those reported as absent are white, and the hatched lines are unknown. *Based on autopsy results of pregnancies terminated at 25 and 22 weeks’ gestation (7). # Patient 6 reported to have rectal bleeding (5).

Despite the involvement of the same gene, the organ systems involved and manifestations within each system contrast significantly from *PMM2*-CDG (Fig. 2). Hypoglycemia and HI have been reported in *PMM2*-CDG but only in up to 2% of large patient cohorts (17, 18). The reported prevalence of kidney and gastrointestinal involvement is substantially lower, and the nature of the system involvement differs (Fig. 2) (2, 3). Nephrotic syndrome and proximal tubulopathy have been reported in *PMM2-*CDG, but also kidney cysts in some (19). Chronic diarrhea and protein-losing enteropathy have been described, but not IBD, whereas liver manifestations, if present, are often more severe, including liver failure and ascites.

**Figure 2:**
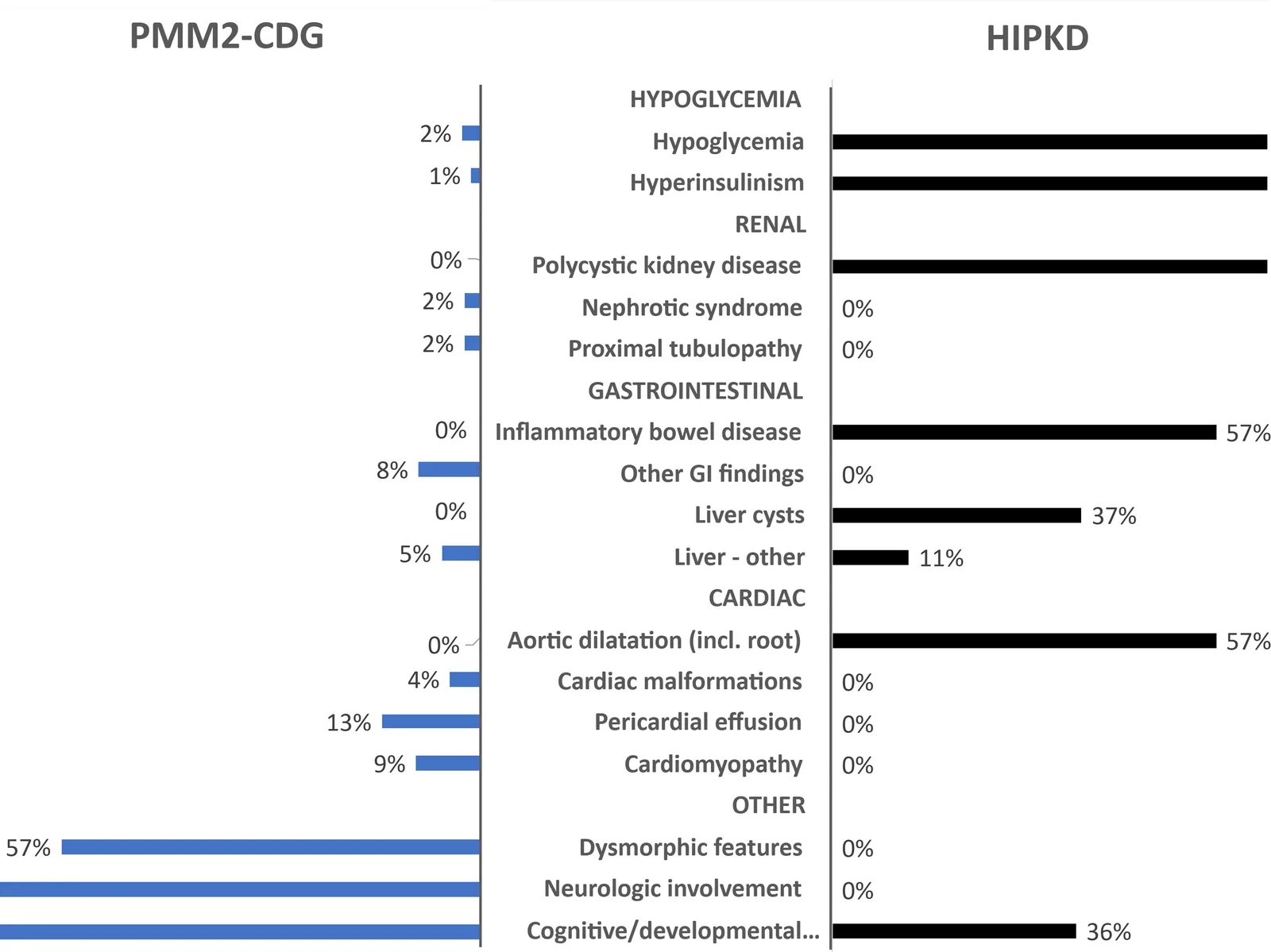
Difference in clinical manifestations of PMM2-CDG vs reported cases of HIPKD. The phenotypic spectrum of HIPKD cases comprised of the patients from this study as well as those previously reported cases (5-7, 16) (dark gray) is contrasted with the phenotype of *PMM2-*CDG (3, 17, 18, 20, 21) (light gray). The antenatal cases (7) were included the “Polycystic kidney disease,” “Liver cysts,” and “Liver—other” categories because of the availability of autopsy data but were excluded from the other categories.

Cardiomyopathy and pericardial effusion have been well described in *PMM2*-CDG cohorts but were not present in our sample. However several patients in this study were found to have aortic dilation at the aortic root and ascending aortic level, although this was not systematically assessed in all patients (20, 21). The contribution of concomitant renal disease and systemic hypertension in the etiopathogenesis of aortic dilation cannot be easily elucidated in view of the small cohort size. Of note, 3/4 patients with aortic dilation were treated with blood pressure-lowering medications, and at last surveillance only requiring a low dose of 1 agent, an angiotensin II receptor blocker. Aortic dilation was mild to moderate and rapid progression was not observed during surveillance. The prevalence and degree of dilation appears excessive when compared to children with other causes of high blood pressure or renal impairment raising the possibility of aortic dilation as part of the spectrum of phenotypic expression, and potentially then exacerbated by hemodynamics and renal dysfunction (22, 23). Aortic dilation associated with polycystic kidney disease has been in reported in GANAB-autosomal dominant polycystic kidney disease, another N-glycosylation defect, suggesting the aortic dilation in our cohort is a feature of the HIPKD spectrum and not simply coincidental (24).

Finally, neurodevelopmental impairment is present in nearly all patients with *PMM2*-CDG, with many having serious involvement. In comparison, the features described here are mild, their direct association with *PMM2* is unclear as hypoglycemia from HI may have contributed to their development, and conditions such as ADHD are prevalent in the general population.

The contrast in features between *PMM2*-CDG and HIPKD illustrates that *PMM2* is clearly a pleiotropic gene by which variants impact multiple different traits in a phenotype (25). Additionally, 9 of the 10 patients in this study had the exact same genotype (c.-167G > T/c.422G > A) despite varied involvement and severity of the HI, renal, gastrointestinal, and cardiac manifestations. The c.422G > A p.(Arg141His) variant affects the enzyme's catalytic site and has an allele frequency of 0.49% (7698/1 570 944) in gnomAD, explaining its high frequency in both CDG and patients with HIPKD (26). However, the promoter variant, which is a European founder variant (27), is absent in the gnomAD database (v 4.1.0 accessed on March 27, 2026) and has not been described in individuals with PMM2-CDG (2, 28).

Thus, the mechanism by which the different manifestations of HIPKD vs PMM2-CDG occur may be related to the regulation of PMM2 expression because all reported individuals with HIPKD have the same promoter variant, c.-167G > T, either homozygous or in trans with a missense or splicing variant (4-7, 16). This promoter variant is in a known binding site for the transcription factor ZNF143. It has been hypothesized that ZNF143 establishes a functional chromatin loop through simultaneous binding of the *PMM2* promoter and specific CTCF (CCCTC-binding factors) sites that can enable spatiotemporal regulation of PMM2 expression (4). Because PMM2 is ubiquitously expressed, it has been hypothesized that tissue specificity may be conferred by tissue-specific transcription factor(s) such as HNF4A; indeed, an increasing degree of gene pleiotropy is correlated with an increased number of protein-protein interactions (25).

Given that the additional manifestations we describe are predominantly structural in nature (renal cysts, gastric antral foveolar hyperplasia, and aortic root dilation), it is plausible that the promoter variant exerts its principal effects during critical windows of organ development, when the *PMM2* promoter is accessible in relevant epithelial and vascular progenitor populations. Alternatively, aortic root dilation was not present at initial assessment and may instead be related to the biomechanical properties of the aorta. Systematic interrogation of publicly available chromatin accessibility datasets (eg, ATAC-seq and histone mark data from fetal kidney, pancreas, gastrointestinal tract, and cardiovascular tissues) could further clarify the developmental regulatory landscape of the *PMM2* promoter, but such analyses are beyond the scope of the present study.

In addition to regulation of gene expression, *PMM1,* a paralogue of *PMM2*, may contribute to pleiotropy (29). *PMM1* has no known disease association, but thermodynamic and statistical modeling suggests that the encoded protein has the capacity to form stable heterodimers with PMM2 providing another theoretical mechanism through which different variants in *PMM2* could result in different manifestations.

The limitations of this study lie in its retrospective nature with incomplete data and variable duration of follow up. For instance, systemic imaging and endoscopy was either not performed or these details were unavailable for the entire cohort. Thus, aortic dilation and/or IBD may be more common in this population. Follow-up duration also varied (Table 1); thus, additional features may develop over time, particularly in the younger patients. Furthermore, the small sample size and ascertainment bias may be influencing our understanding of the phenotype as the patient cohort was preselected for HI and PKD, whereas 2 patients reported by Dorval et al with the same genotype as those in this study had renal manifestations only with no history of hypoglycemia (7). We also did not perform any functional validation of *PMM2* promoter activity in patient-derived tissues, although previous work has demonstrated impaired promoter function with the c.-167G > T variant (4).

Despite these limitations, this study details a broader degree of multisystem involvement than previously described associated with the promoter variant in *PMM2* (Fig. 1), expanding our understanding of the associated phenotype and its distinction from the features seen in PMM2-CDG. The development of IBD and aortic dilation underscores the need for clinicians to enquire about gastrointestinal symptoms in these patients as well as consider cardiovascular surveillance with echocardiogram. Based on these findings, we suggest baseline echocardiography and targeted gastrointestinal evaluation in individuals with HIPKD, in addition to standard endocrine and kidney surveillance.

## Data Availability

Original data generated and analyzed during this study are included in this published article.
